# Supplementary material for: Precision-induced localized molten liquid metal stamps for damage-free transfer printing of ultrathin membranes and 3D objects
Source: Nat Commun. 2024 Oct 13;15:8839. doi: 10.1038/s41467-024-53184-7 (PMC11471857; doi:10.1038/s41467-024-53184-7)
Supplement: Supplementary file 1 — Supplementary Information [file 41467_2024_53184_MOESM1_ESM.pdf]

Supplementary Information

Precision-Induced Localized Molten Liquid Metal Stamps for Damage-Free Transfer Printing of Ultrathin Membranes and 3D Objects

Chuanqian Shi[1,2,3], Jing Jiang[3], Chenglong Li[3], Chenhong Chen[3], Wei Jian[4] and Jizhou Song[3,5,6]*

[1]Center for Mechanics Plus under Extreme Environments, School of Mechanical Engineering & Mechanics, Ningbo University, Ningbo 315211, China
[2]Key Laboratory of Impact and Safety Engineering, Ministry of Education, Ningbo University, Ningbo 315211, China
[3]Department of Engineering Mechanics, Soft Matter Research Center, Key Laboratory of Soft Machines and Smart Devices of Zhejiang Province, State Key Laboratory of Brain-Machine Intelligence, Zhejiang University, Hangzhou 310027, China
[4]Zhejiang-Italy Joint Lab for Smart Materials and Advanced Structures, School of Mechanical Engineering & Mechanics, Ningbo University, Ningbo 315211, China
[5]Department of Rehabilitation Medicine, The First Affiliated Hospital School of Medicine, Zhejiang University, Hangzhou 310003, China
[6]Institute of Flexible Electronics Technology of THU, Zhejiang, Jiaxing 314000, China

*To whom correspondence should be addressed. jzsong@zju.edu.cn

The PDF file includes:

Supplementary Note 1: Calculations of adhesive force and shear strain
Supplementary Note 2: Observation and analyses of gallium oxide
Supplementary Note 3: Laser heating temperature gradient analysis
Supplementary Note 4: Molecular dynamics (MD) simulation methods
Supplementary Figs. 1 to 25

Supplementary Note 1: Calculations of adhesive force and shear strain

In the adhesive force analysis shown in Figure 2C, the calculation of adhesive force requires solving for the liquid Ga profile, taking into account the specified liquid volume and contact angles. A variable transformation changes the original Young–Laplace equation into the following system of ordinary differential equations with the arc length as the independent variable[1]:

$$\frac{\mathrm{d}x}{\mathrm{d}s} = \cos\varphi , \tag{S1}$$

$$\frac{\mathrm{dz}}{\mathrm{d}s} = \sin\varphi , \tag{S2}$$

$$\frac{\mathrm{d}\varphi}{\mathrm{d}s} = \frac{\Delta P}{\gamma} - \frac{\sin\varphi}{x} , \tag{S3}$$

where x and z are the coordinates of the axisymmetric liquid bridge, φ is the angle between the local tangent of the liquid surface and the horizontal axis, and s is the arc length of the liquid profile, as indicated in red in Figure 2C. For given values of liquid volume V, contact angles θ_1 and θ_2, a unique liquid surface profile can be obtained by simultaneous integration of the above equation system. We treat Equations (S1)–(S3) as a typical initial value problem: the red dot in x axis is assumed to be the point where the liquid profile meets the cylinder tip, as indicated in Figure 2C. The initial values of the point are: $x(0) = x_1$, $\mathrm{z}(0) = 0$, $\varphi(0) = \pi - \theta_1$. Also, the calculated liquid volume V and the θ_2 contact angle should match the prescribed value, respectively, that are

$$V = \int_0^h \pi \cdot x^2 dz , \tag{S4}$$

and

$$\varphi\big|_{z=h} = \theta_2 . \tag{S5}$$

The initial guesses of the values of h and θ_1 are made at the start of integration, and a numerical iteration scheme is adopted to determine h and θ_1 by two conditions in Equation (S4) and

Equation (S5). Once the values of h and θ_1 are determined, the liquid droplet profile and the adhesive force can be calculated, respectively. We take V to be 0.055cm^3, θ_2 to be 100 degrees based on the experimental data in Supplementary Figure 7, and take the surface tension of liquid Ga to be 680 mN/m[2].

In the shear strain calculation, we consider the thickness of the silicon film h_m to be 600 nm, Young's modulus and Poisson ratio of silicon film to be 160 GPa and 0.4, respectively, which can be used to calculate lame contents of the silicon film μ_m and λ_m. The Young's modulus and Poisson's ratio were 2 MPa and 0.49 for PDMS, respectively.

Supplementary Note 2: Observation and analyses of gallium oxide

(1) Optical observation: Although gallium oxide nanomembrane is transparent, the existence of gallium oxide can be observed by the surface luster and wrinkles of gallium metal droplets due to light scattering of Ga_2O_3 (Supplementary Figure 6A) [3–5]. In Supplementary Figure 6, we have incorporated additional experiments utilizing optical magnification to observe the surface sheen of liquid gallium during the preload process. This allows us to detect the rupture of the Ga_2O_3 film to some extent. In our experiments, we have observed subtle differences in the surface appearance between the liquid-phase Ga droplets encapsulated by the Ga_2O_3 nanomembrane and the bare liquid gallium. Upon the rupture of Ga_2O_3, a metallic sheen is revealed on the surface of the liquid gallium. During the preload process, we found that when contacting planar films or objects with smooth surfaces, there is barely observable rupture of the Ga_2O_3 nanomembrane (Supplementary Figure 6B). In cases involving irregular or sharply angled objects, the Ga_2O_3 nanomembrane may experience a puncture, exposing the metallic sheen of the liquid gallium (Supplementary Figure 6C). However, visual observations are imprecise. Investigating the systematic aspects of Ga_2O_3 film rupture during the preload process involves considering factors such as the magnitude of preload force, the shape of objects, and the impact of effective contact area on the results. Moreover, due to the rapid generation of

Ga_2O_3[6], real-time observation methods are necessary to capture the instantaneous rupture of the Ga_2O_3 film. Therefore, addressing the rupture of the Ga_2O_3 film during the preload process is a complex and systematic issue that requires a thorough investigation. We plan to conduct further research in subsequent work to explore this aspect comprehensively.

(2) Analyses of gallium oxide: We analyzed the extent of gallium oxidation of a 3 mm diameter Ga stamp over different cycles of heating and cooling using high-resolution x-ray photoelectron spectroscopy (XPS, AXIS ULTRA DLD, Kratos Analytical Co. Ltd.)[7], whose results show five samples (Figures S7A). Previous study demonstrates the oxide nanofilm initially forms with a thickness of one unit cell layer, and then grows in the ambient atmosphere to a maximum thickness of up to 3 nm[3]. As the oxide layer thickness increases, the pure Ga metal content (centered at approximately 1116.7 eV) in the Ga 2*p* spectral region shows significant differences. Specifically, the relative amount of pure Ga 2*p*3 metal decreases from 28.81% in the original test (Fig. S7A, top left) to 24.29% after 15 cycles of heating and cooling (Fig. S7A, bottom right). Conversely, the amount of Ga 2*p*3 oxide and suboxide increases in relative proportion from 71.19% (Fig. S7A, top left) to 75.71% (Fig. S7A, bottom right). This indicates that additional gallium oxide forms in the air as the heating and cooling cycles increase. By approximately the 5th cycle, the gallium oxide growth stabilizes, and the relative proportions of gallium oxide in 5th (75.34%), 9th (75.5%), and 15th cycle (75.71%) remain almost unchanged.

Supplementary Note 3: Laser heating temperature gradient analysis

In the description of the laser heating process, we stated that the laser heats the solid Ga surface with an attached picked-up Si platelet, in turn, heats the interface and the Ga stamp in the vicinity. Supplementary Figure 1B shows the schematic of the model consisting of a 2 μm-thick Si platelet with a diameter of 400 μm and a Ga stamp with a diameter of 2.3 mm and a height of 1.85 mm. The bottom surface of the Ga stamp (in Supplementary Figure 1B) is fixed,

and the bottom surface of the Si platelet is constrained to move with the top surface of the Ga stamp. Other surfaces in this model are free to move. We used a COMSOL®[1] finite element model with the top surface including the stamp–ink interface acting as the heat source, and the exposed surfaces of the Si platelet and Ga stamp lose heat to the surroundings by convection. The strength of the heat source is varied, and the corresponding steady state temperatures are computed. The model uses 8,769 nodes to perform a transient heat transfer analysis in COMSOL 6.0 for run intervals up to 200 ms (our typical laser pulse times is 200 ms) with the silicon platelet, Ga, and surroundings initially at 20.3 ℃. Fig. S1B shows the results of one run, in which the power of the laser system 13 W at the top surface area 3.1 mm^2 of heat is input into the system in 200 ms, and the calculated power density is 0.82 W/mm^2. The thermal conductivity of gallium and silicon is 37.68 W/ (m·K) and 152 W/ (m·K), respectively. From this simulation, one can see that the temperatures reached in the system are about 36.2 °C, slightly higher than the melting point of metal Ga 29.76 °C, sufficient to cause localized melt without damaging the ink.

Supplementary Note 4: Molecular dynamics (MD) simulation methods

Molecular dynamics (MD) simulations were performed to investigate the mechanical behaviors between the Gallium (Ga)-based stamp and the silicon wafer during the contact process. The Ga-based liquid metal contained two components: the Gallium oxide (Ga_2O_3) film and the liquid Gallium core. During the modeling, the film model of Ga_2O_3 in spherical-shell structure was constructed with the diameter of 12 nm and the thickness of 2 nm. The Ga liquid metal with 8 nm diameter was added into the inner of the Ga_2O_3 shell to create the Ga-based stamp. The nanoscale crystalline silicon with the dimensions of 5.43 × 5.43 × 3.26 nm^3 and $\alpha = \beta = \gamma = 90°$ was constructed as the wafer under the Ga-based stamp. The initial model was shown in Supplementary Figure 15A, and the configuration was provided in Supplementary Data 1. The Tersoff based interatomic potential for Ga liquid metal and silicon wafer, combined

with a Born-Mayer-Huggins type expression for Ga_2O_3 film was applied to describe the interactions among metallic atoms in this system, which has been verified and evaluated for the related metallic systems[8-10]. The energy minimization was performed to obtain a stable structure. The system was then equilibrated under the NVT ensemble (constant number of particles, volume and temperature) at a constant temperature of 300 K for 1 ns to achieve an equilibrium state. After full equilibrium, MD simulations for the contact process were performed by moving the Ga-based stamp towards the silicon wafer until the stamp was deformed with the strain of 40%. The final configuration was provided in Supplementary Data 2. The non-periodic boundary conditions were applied in all three dimensions. To analyze the effect of the crystallization process of gallium metal, the Ga-based stamp was cooled down to 50 K when it has already been compressed 40% on the silicon wafer. The corresponding configuration was provided in Supplementary Data 3. The local atomic strain distribution with the reference to the relaxed system before the contact was captured to analyze the deformation status in the system moving during the contact process.

Supplementary References

1. Qian, J. & Gao, H. Scaling effects of wet adhesion in biological attachment systems. *Acta Biomaterialia* **2**, 51–58 (2006).
2. Abbaschian, G. J. Surface tension of liquid gallium. *Journal of the Less Common Metals* **40**, 329–333 (1975).
3. Wang, D., Wang, X. & Rao, W. Precise Regulation of Ga-Based Liquid Metal Oxidation. *Acc. Mater. Res.* **2**, 1093–1103 (2021).
4. Hou, Y. *et al.* Coloration of Liquid-Metal Soft Robots: From Silver-White to Iridescent. *ACS Appl. Mater. Interfaces* **10**, 41627–41636 (2018).
5. Zhao, J., Li, H., Bi, X. & Dai, H. Rapidly reversible discoloration of liquid metal by contact or separation. *Materials Chemistry and Physics* **291**, 126726 (2022).

6. Palleau, E., Reece, S., Desai, S. C., Smith, M. E. & Dickey, M. D. Self-Healing Stretchable Wires for Reconfigurable Circuit Wiring and 3D Microfluidics. *Advanced Materials* **25**, 1589–1592 (2013).

7. Sun, L. *et al.* All-solution-processed ultraflexible wearable sensor enabled with universal trilayer structure for organic optoelectronic devices. *Sci. Adv.* **10**, eadk9460 (2024).

8 Nord, J., Albe, K., Erhart, P. & Nordlund, K. Modelling of compound semiconductors: analytical bond-order potential for gallium, nitrogen and gallium nitride. *J. Phys.: Condens. Matter* **15**, 5649-5662 (2003).

9 Pun, G.P. Purja & Mishin, Y. Optimized interatomic potential for silicon and its application to thermal stability of silicene. *Phys. Rev. B* **95**, 224103 (2017).

10 Petkov, A., Mishra, A., Pomeroy, J.W. & Kuball, M. Molecular dynamics study of thermal transport across Ga2O3–diamond interfaces. *Appl. Phys. Lett*. **122**, 031602 (2023)

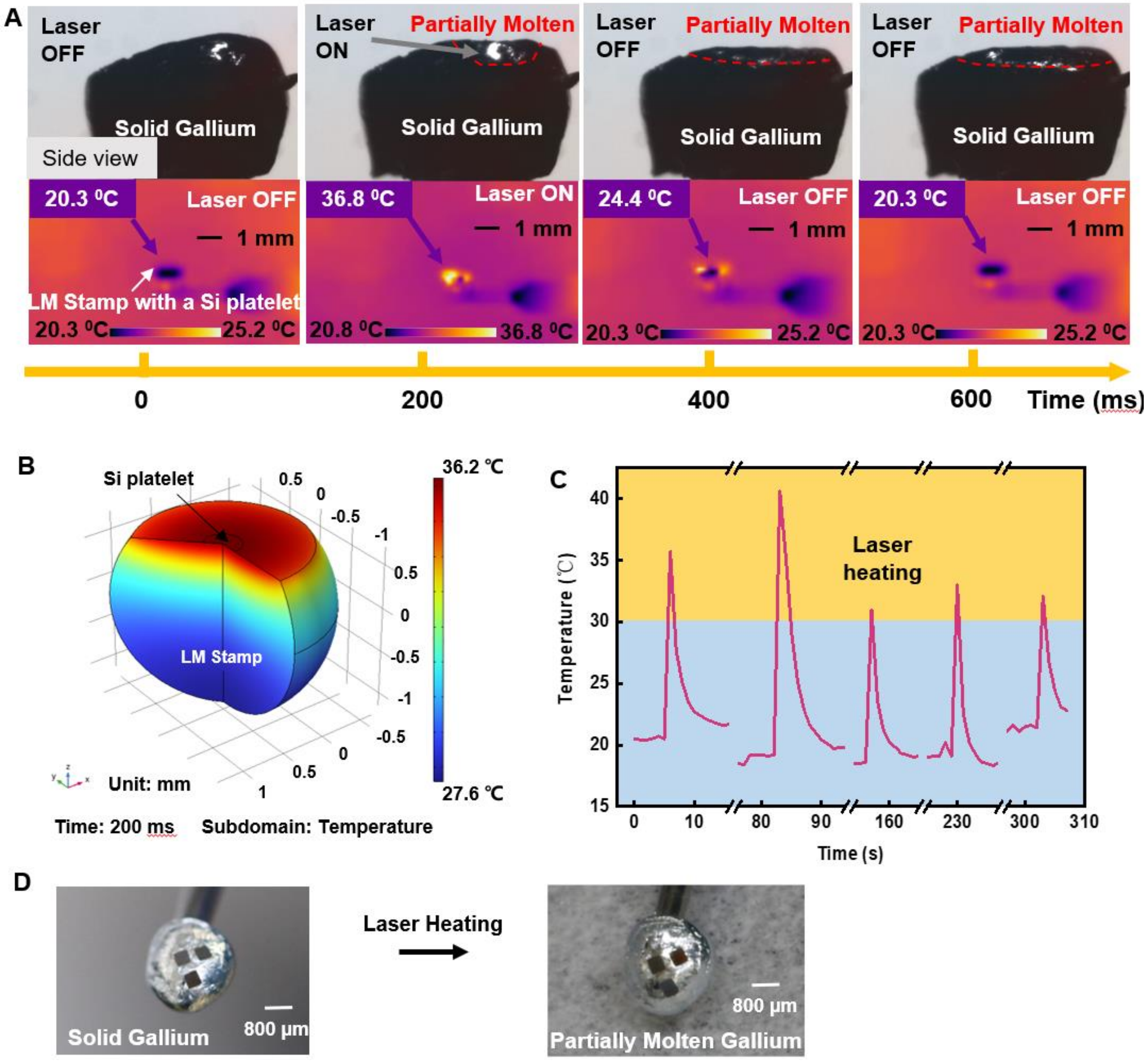

Supplementary Figure 1. (A) The high-speed photographs and thermograms of gallium (Ga) before and after laser heating. (B) Finite element model of the stamp and ink showing temperature gradient in the stamp and attached ink. (C) The temperature curves of Ga before and after laser heating for 6 times. (D) The relative positions of the Si array on solid and localized molten gallium surfaces before and after laser heating, respectively. Source data are provided as a Source Data file.

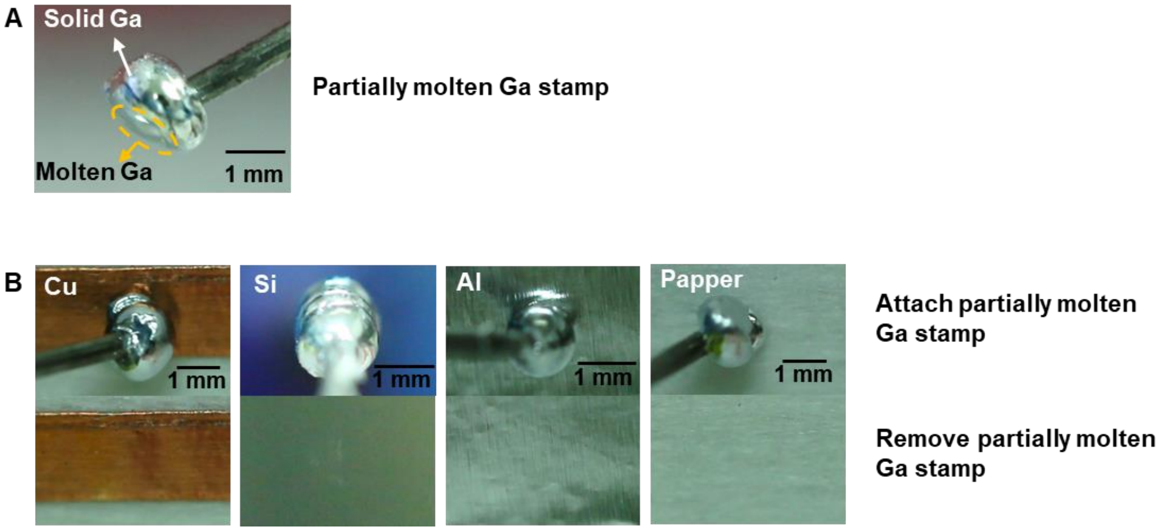

Supplementary Figure 2. The localized molten state of metal Ga stamp. (A) The coexistence of liquid and solid phases of Ga. (B) The localized molten Ga easily peeled from the object's surfaces. The contact of the localized molten stamp to various materials (first row) and peeled easily (second row).

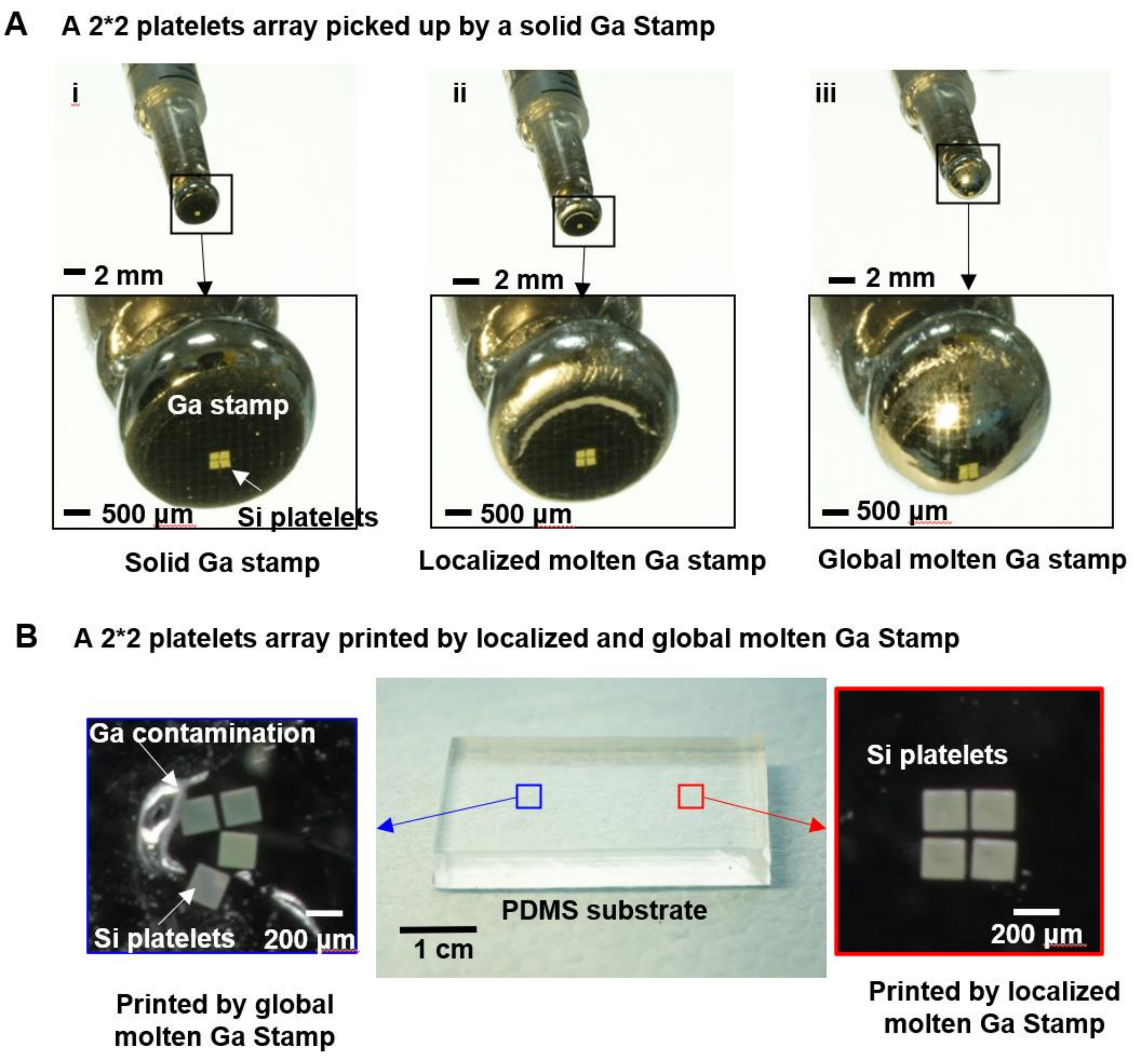

Supplementary Figure 3. Comparison of localized and global molten Ga stamp transfer printing methods. (A) Sequential images illustrating the transfer printing process of a 2 × 2 square silicon platelet array. (B) Images of the silicon platelet arrays printed on PDMS surfaces by global molten method (left with a blue solid line frame) and localized molten method (right with a red solid line frame).

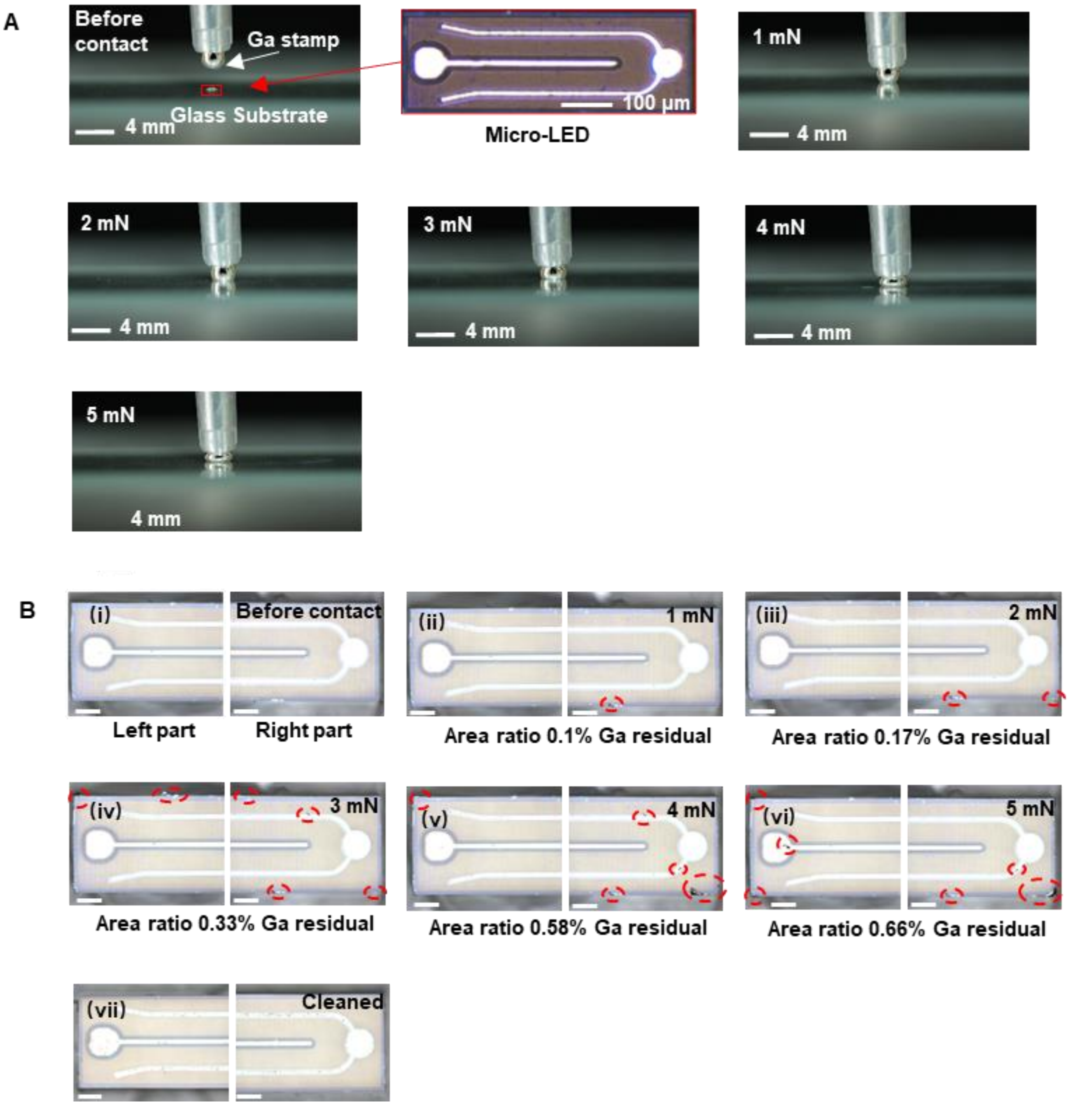

Supplementary Figure 4. (A) photos of liquid gallium stamps (diameter ~ 2 mm) contacting micro-LEDs (with dimensions of 600 μm×200 μm×2 μm) under five different preload conditions. (B) Microscopic images of a micro-LED under different conditions, such as clean, alcohol-cleaned, and gallium residual under different preload (marked with red dotted lines).

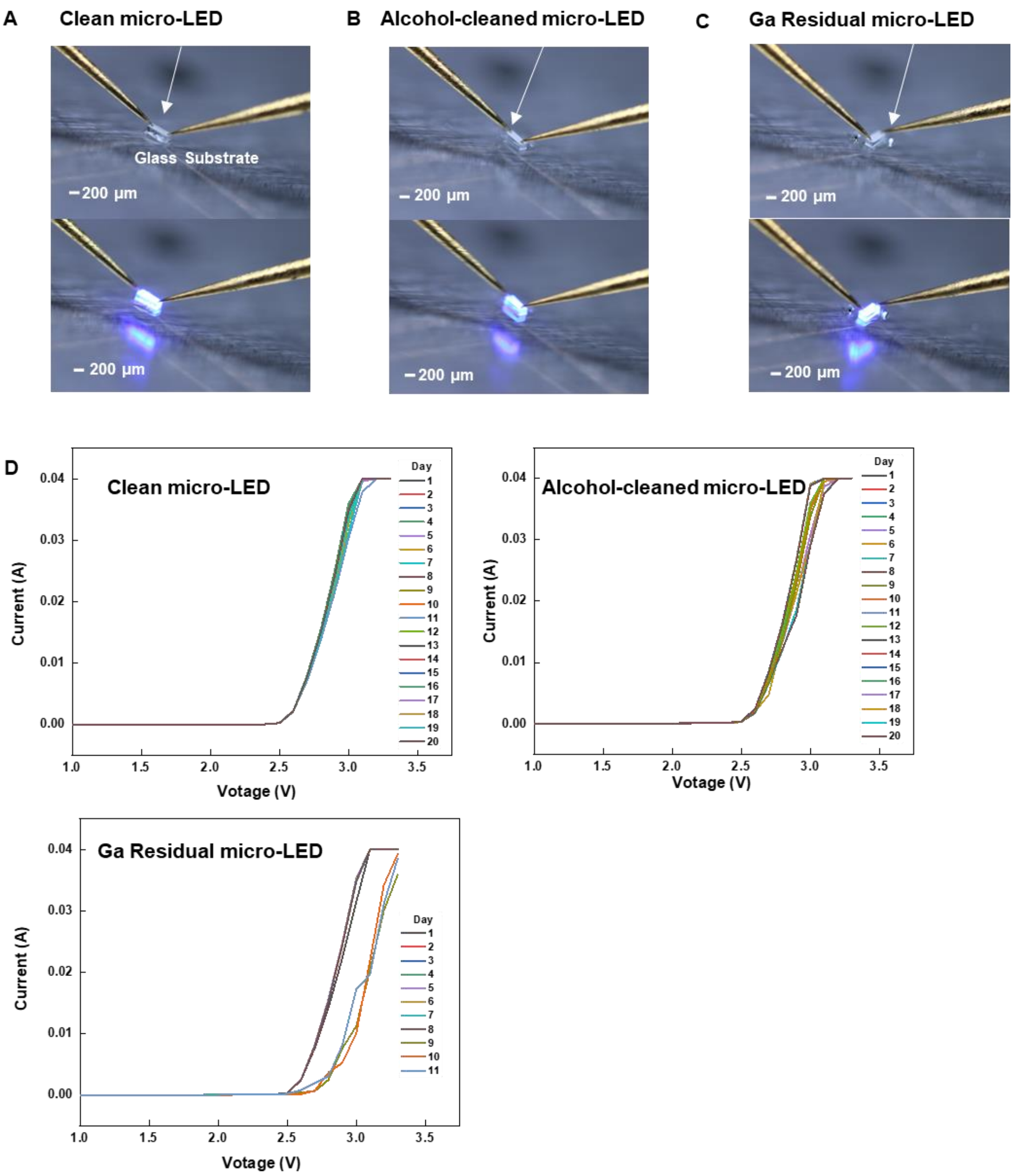

Supplementary Figure 5. Light-emitting capability during proper function for micro-LEDs under different conditions: (A) clean, (B) alcohol-cleaned, and (C) contaminated. (D) Voltage-current curves over several days of operation for micro-LEDs under different conditions. Source data are provided as a Source Data file.

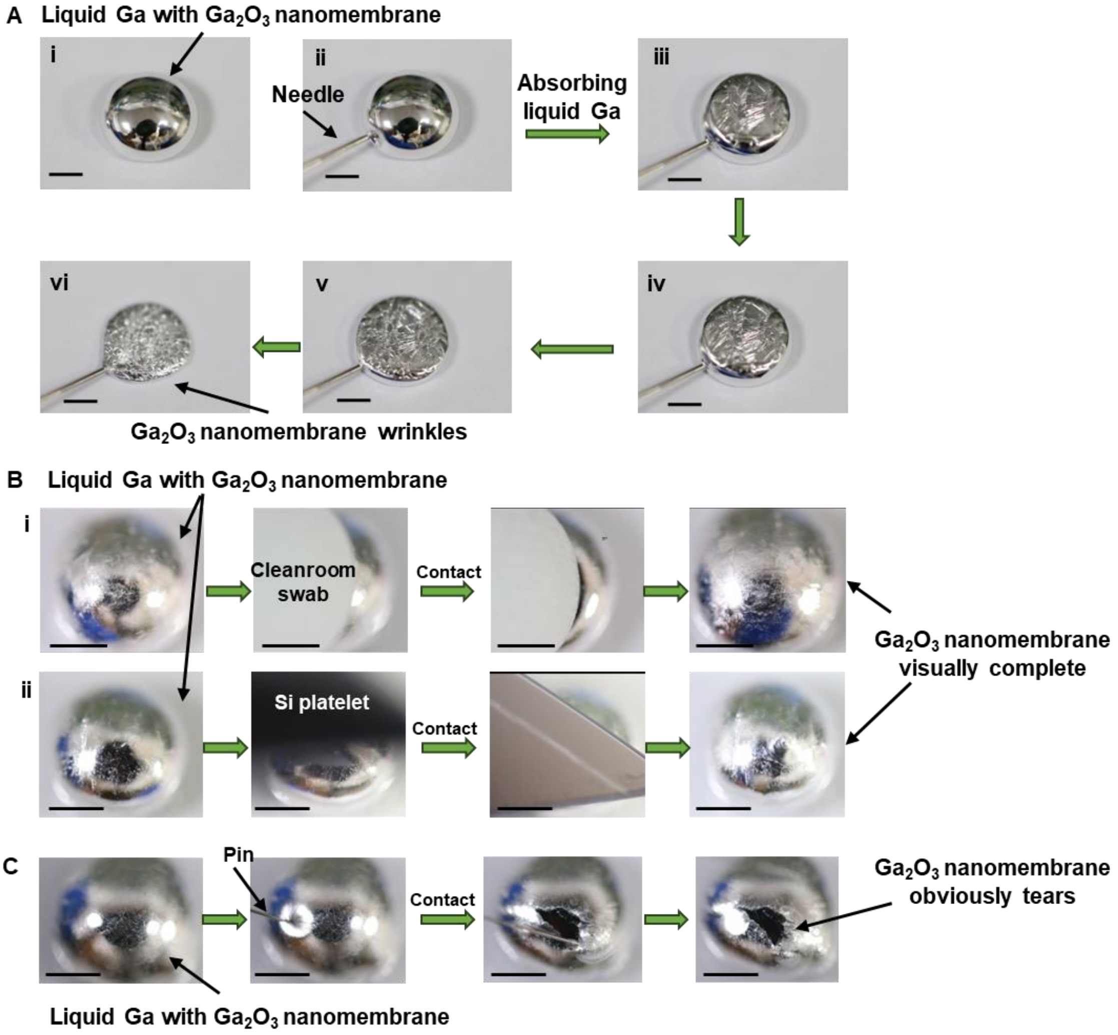

Supplementary Figure 6. (A) Surface oxidation of liquid Ga. (i) A droplet of liquid gallium, where surface oxidation is not readily apparent from a distance. (ii)-(vi) Injection and extraction of gallium metal using a syringe needle. The Ga_2O_3 film on the surface of liquid gallium exhibits wrinkles and a noticeable decrease in surface luster due to droplet reduction and surface instability. (B) Close-up of liquid Ga surface with a noticeable decrease in surface luster due to Ga_2O_3. Interaction with a cleanroom swab (i) and Si platelet (ii), respectively, demonstrated no significant change in surface luster. (C) Rupture of the Ga_2O_3 nanomembrane. Stabbing the liquid gallium droplet with a pin, revealing the rupture of the Ga2O3 nanomembrane and exposing the metallic sheen beneath. Scale bars: 2 mm.

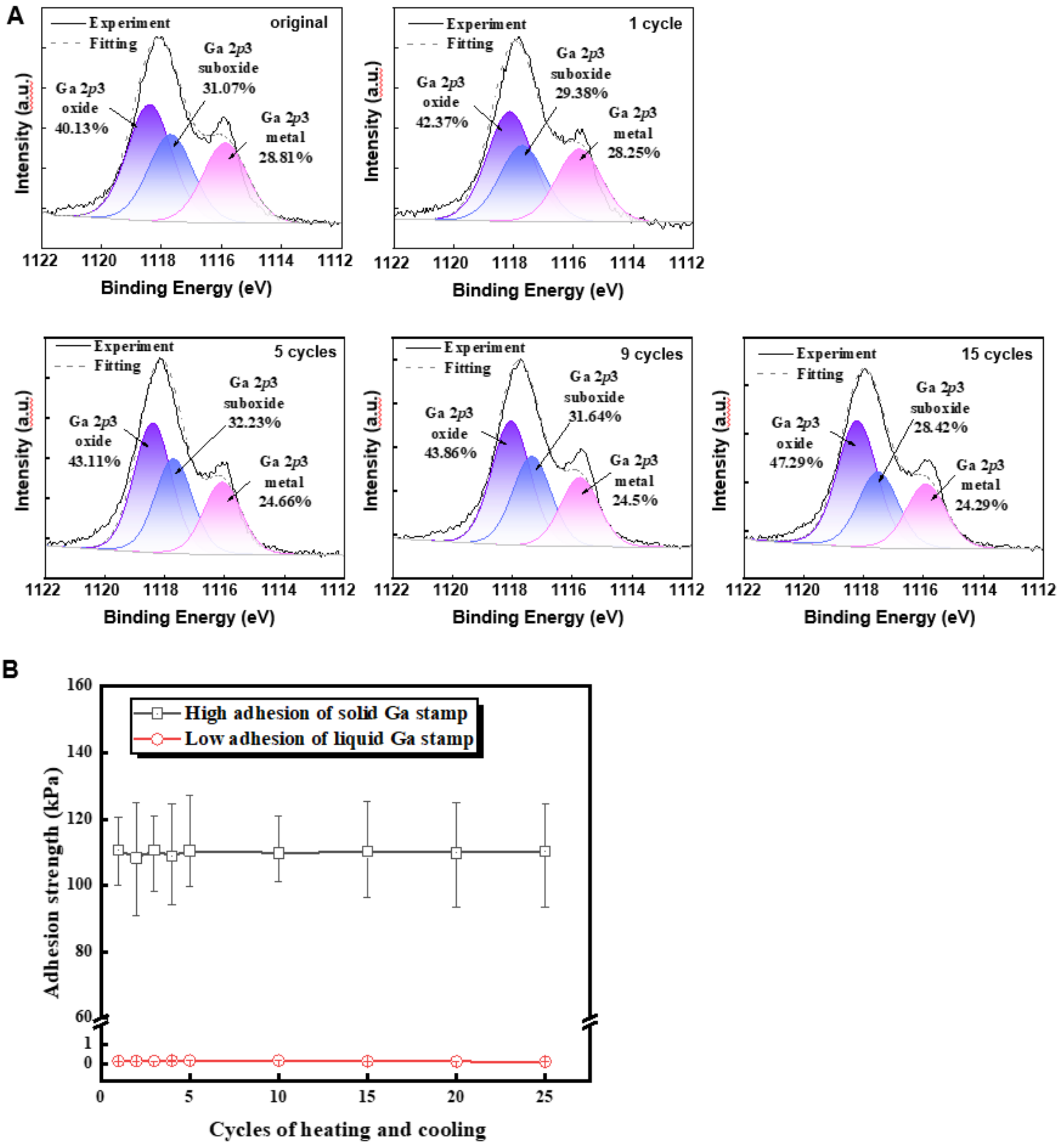

Supplementary Figure 7. (A) XPS spectra of Ga 2*p* of the Ga stamp over different cycles of heating and cooling. (B) The high and low adhesion strength of Ga stamp over different cycles of heating and cooling. The standard deviation is based on 3 repeated experiments. Source data are provided as a Source Data file.

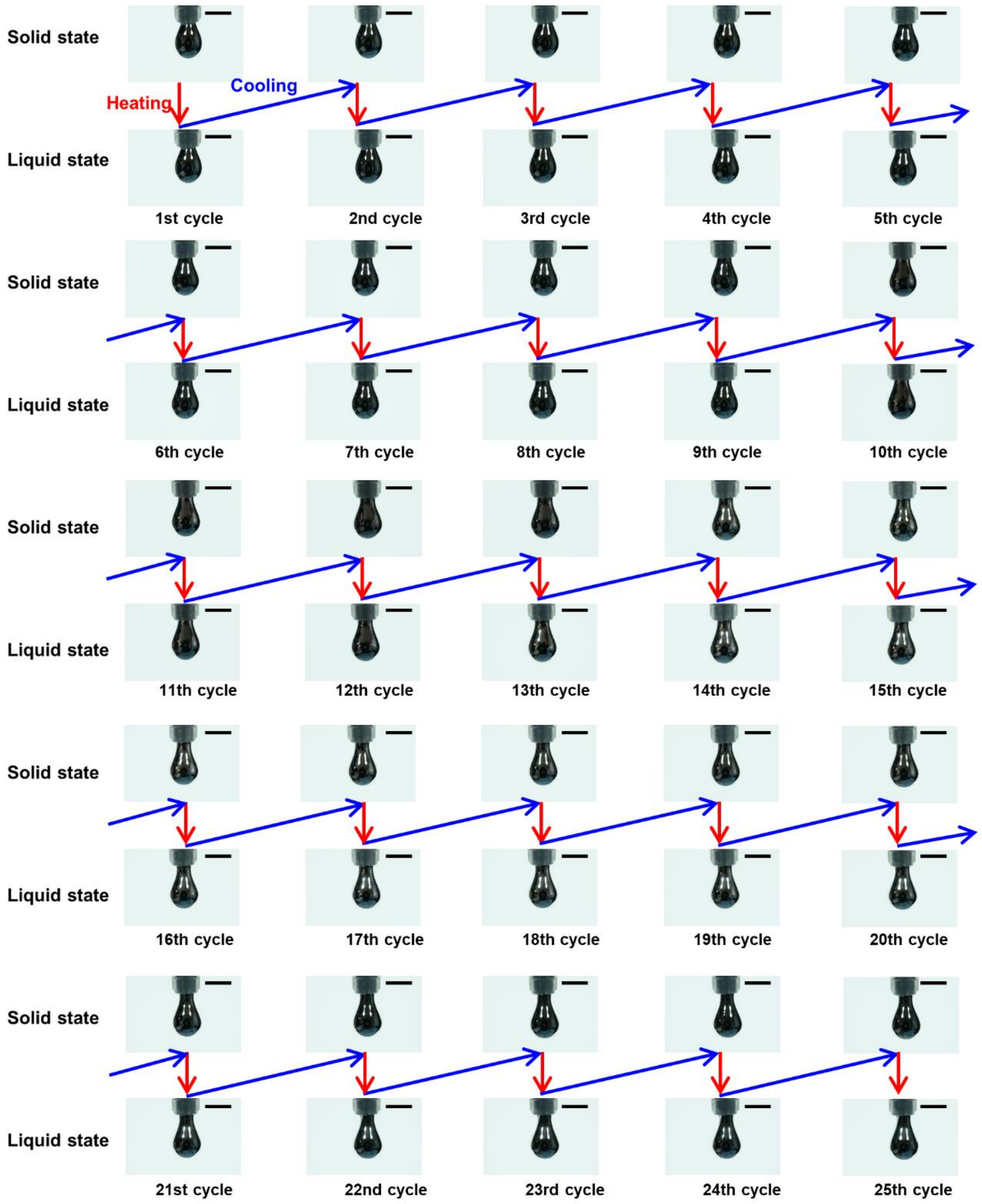

Supplementary Figure 8. The shape of the Ga droplet can remain consistent over 25 cycles of heating and cooling. Scale bars: 4 mm.

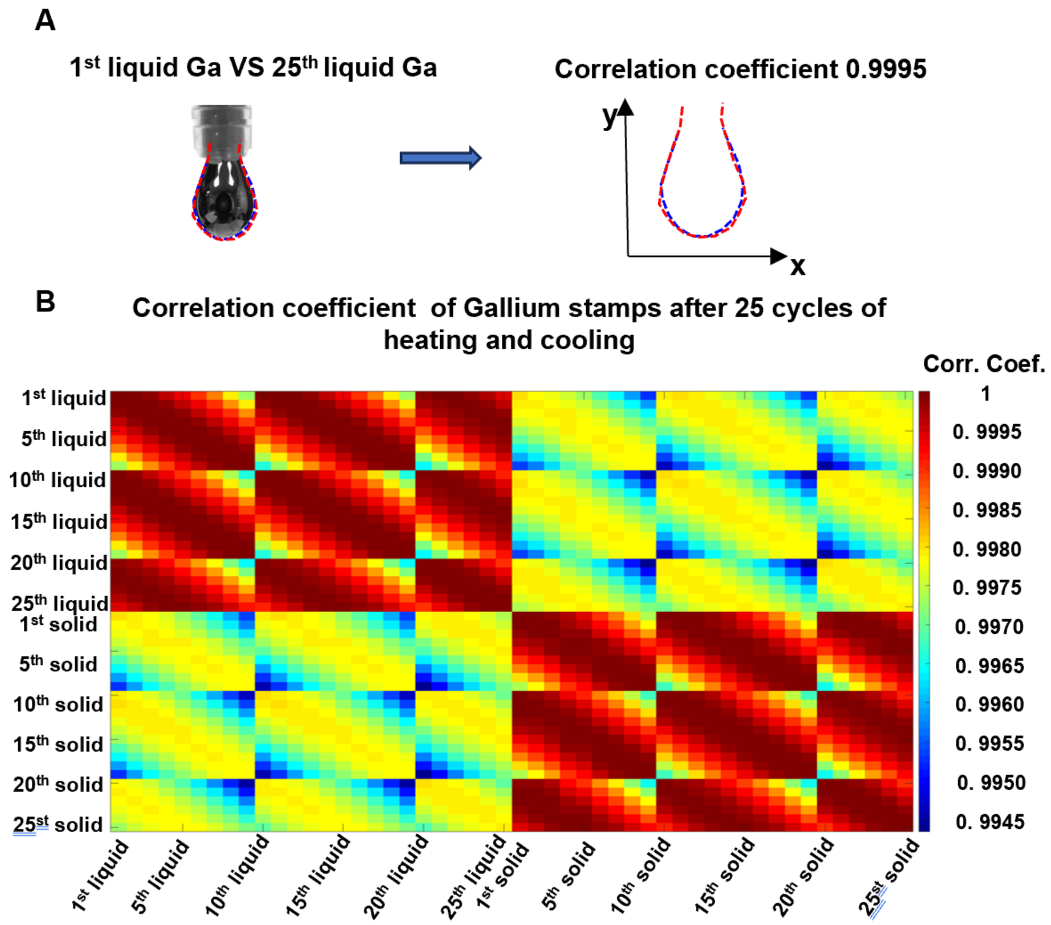

Supplementary Figure 9. (A) The overlapping outlines of the liquid gallium droplet from the first and last heating cycles. (B) The heatmaps of consistency coefficients for both solid and liquid gallium stamps. A relevant code has been uploaded to Supplementary Data 4.

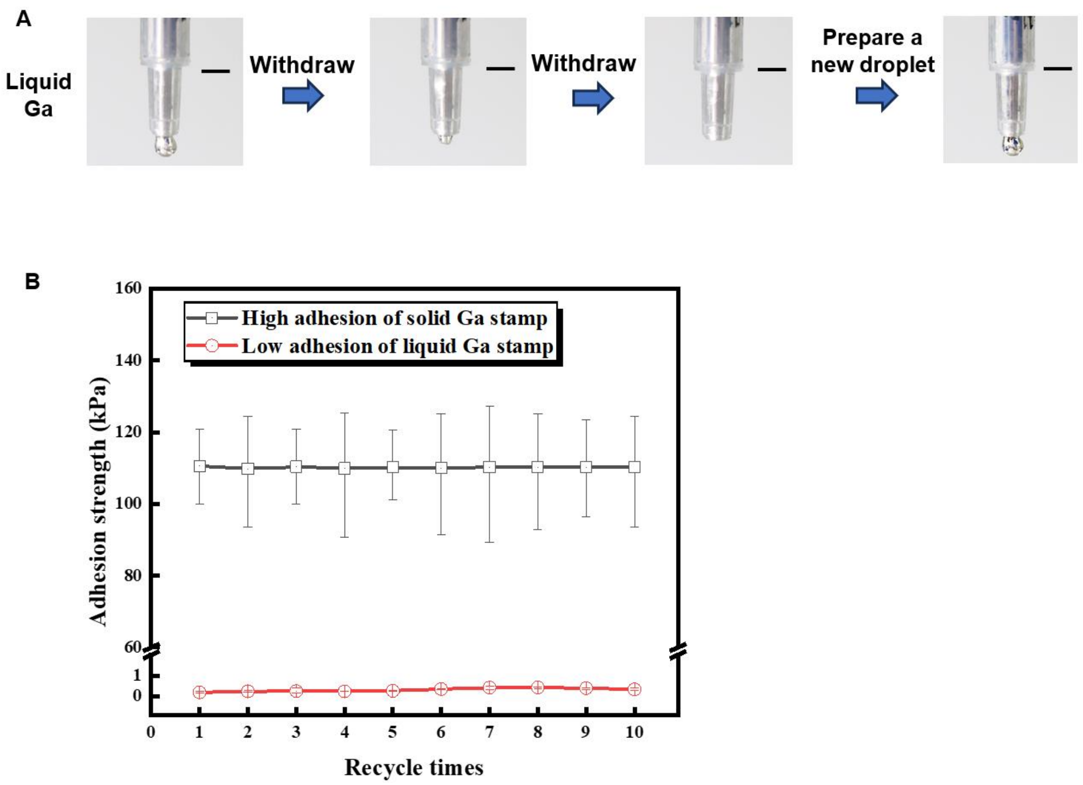

Supplementary Figure 10. (A) The recycled liquid Ga droplet is withdrawn and prepared by a syringe. Scale bars: 4 mm. (B) The high and low adhesion strength of Ga stamp over 10 recycle times. The standard deviation is based on 3 repeated experiments. Source data are provided as a Source Data file.

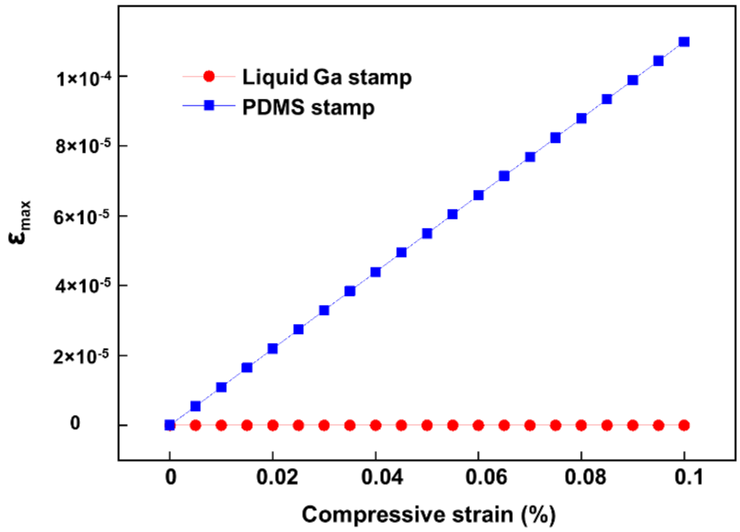

Supplementary Figure 11. The maximum shear strain in silicon film versus the compressive strain applied from PDMS stamp and Liquid Ga stamp. Source data are provided as a Source Data file.

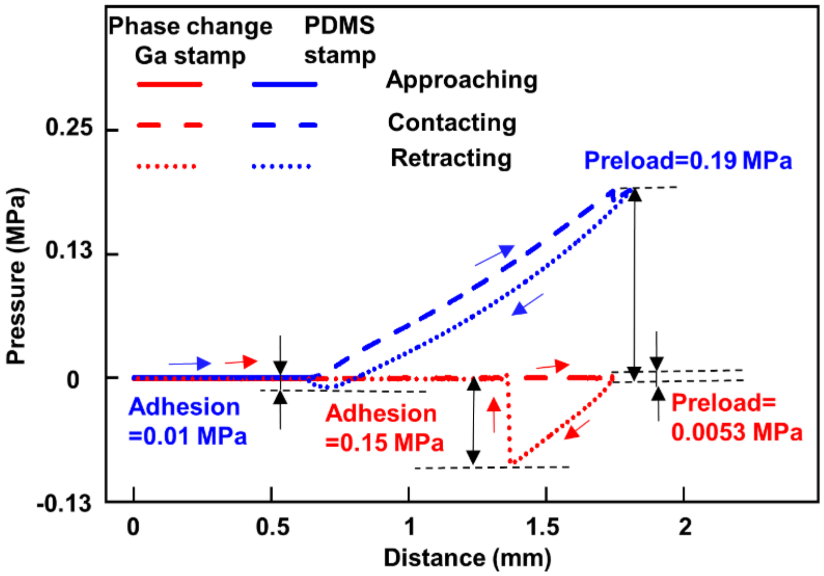

Supplementary Figure 12. Typical pressure-displacement curves for measuring the adhesion strength of the phase change Ga stamp and PDMS stamp. Source data are provided as a Source Data file.

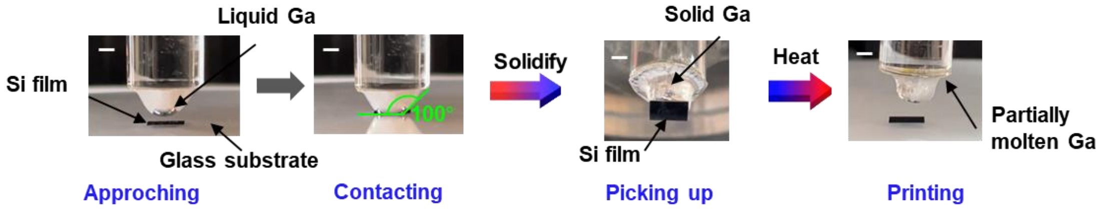

Supplementary Figure 13. Optical images of the picking up and printing process of a Si film. Scale bars: 2 mm.

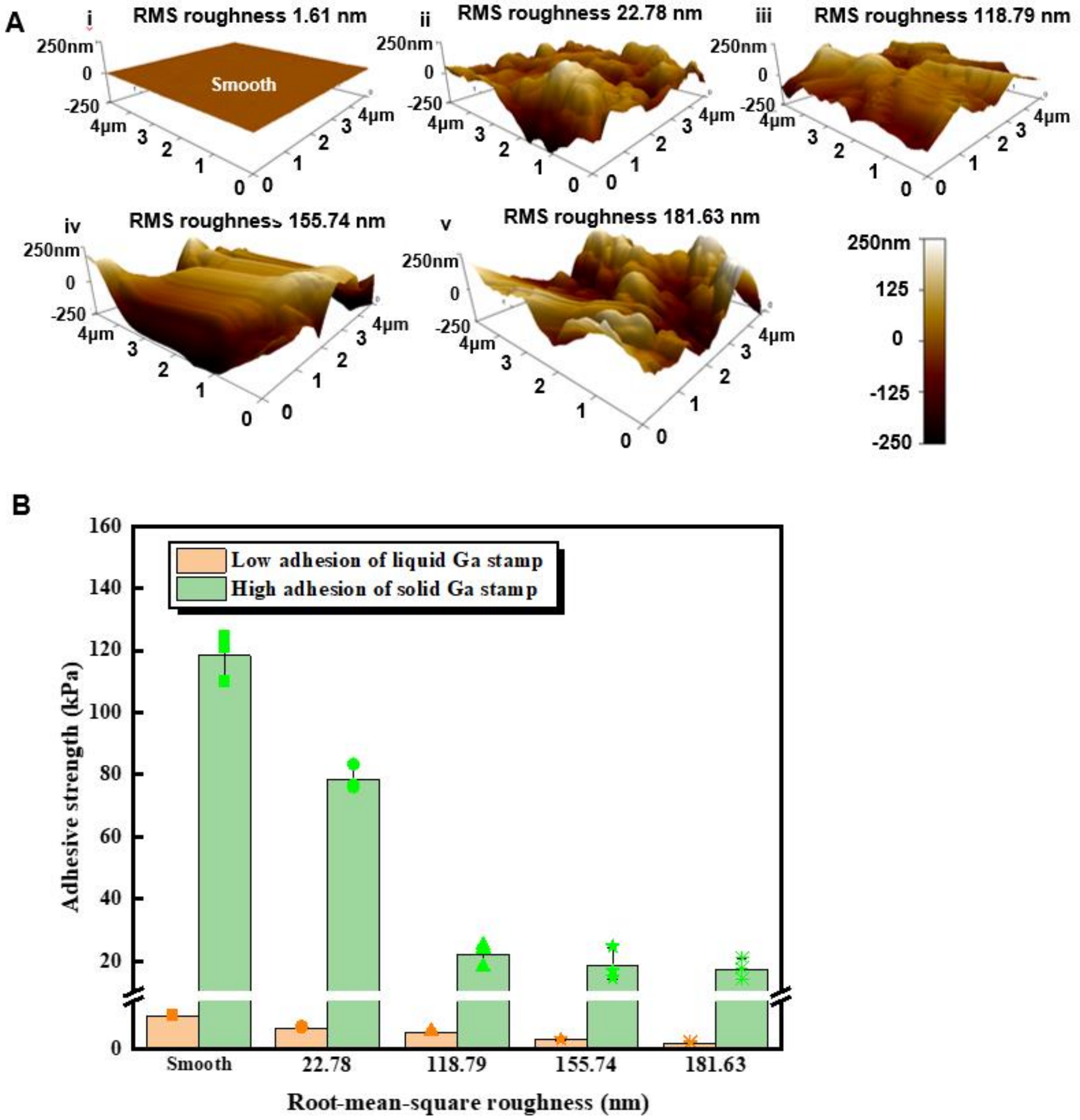

Supplementary Figure 14. (A) Atomic force microscope images of smooth and rough glass surfaces with varying roughness. The root-mean-square roughness values are as follows: smooth glass at 1.61 nm (i), and rough glass surfaces at 22.78 nm (ii), 118.79 nm (iii), 155.74 nm (iv), and 181.63 nm (v). (B) Variation in adhesion strength due to surface roughness. As the roughness of the ink increases, the adhesion strength of the gallium stamp decreases. The standard deviation is based on 3 repeated experiments. Source data are provided as a Source Data file.

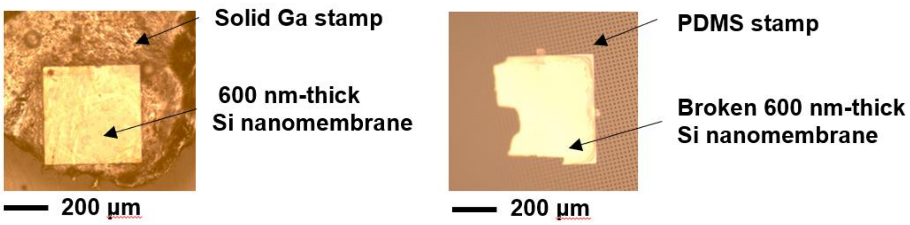

Supplementary Figure 15. 600 nm thick Si nanomembrane transferred intactly by the PLMT method (left) and broken by the traditional PDMS stamp (right).

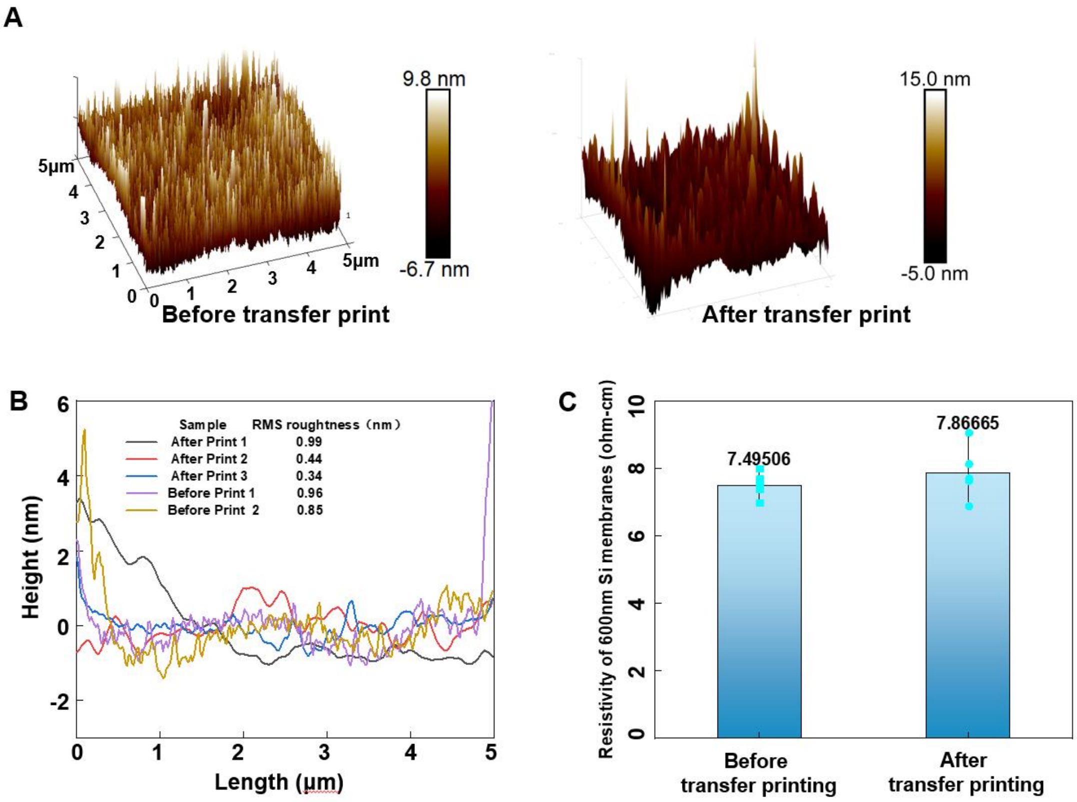

Supplementary Figure 16. (A) Atomic force microscope images of a 600 nm Si membrane before (left) and after (right) the transfer print process. (B) The surface roughness data and the root-mean-square roughness values of samples before and after transfer print process. (C) The resistivity values of the Si nanomembrane before and after transfer print process. The standard deviation is based on 5 repeated experiments. Source data are provided as a Source Data file.

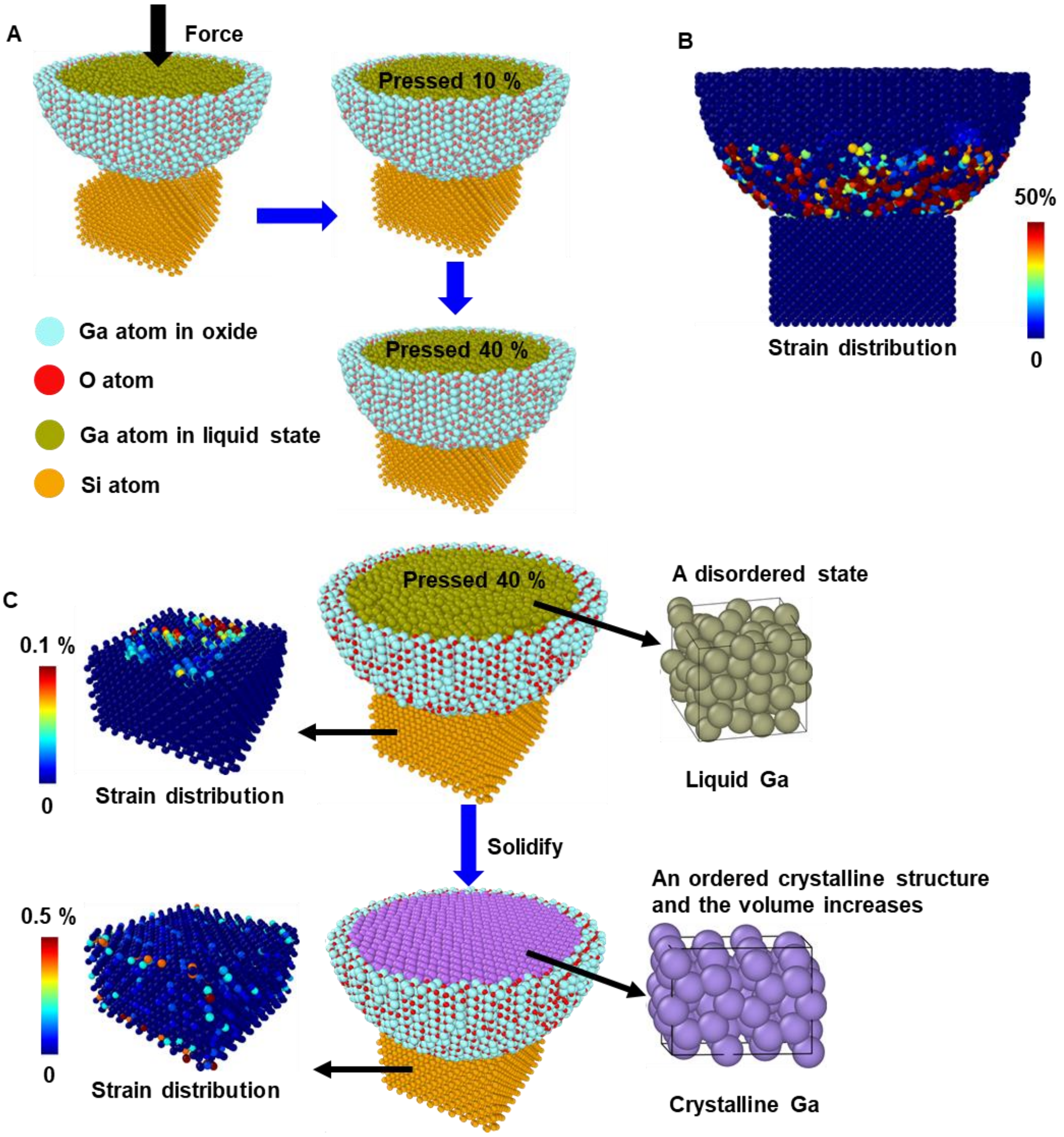

Supplementary Figure 17. (A) The atomistic structure and contact process of the Ga-based stamp on the nanoscale silicon wafer. (B) The local atomic strain distribution of the whole system. (C) The atomistic structure and contact process with the local atomic strain distribution when Ga was transformed from liquid state to solid state.

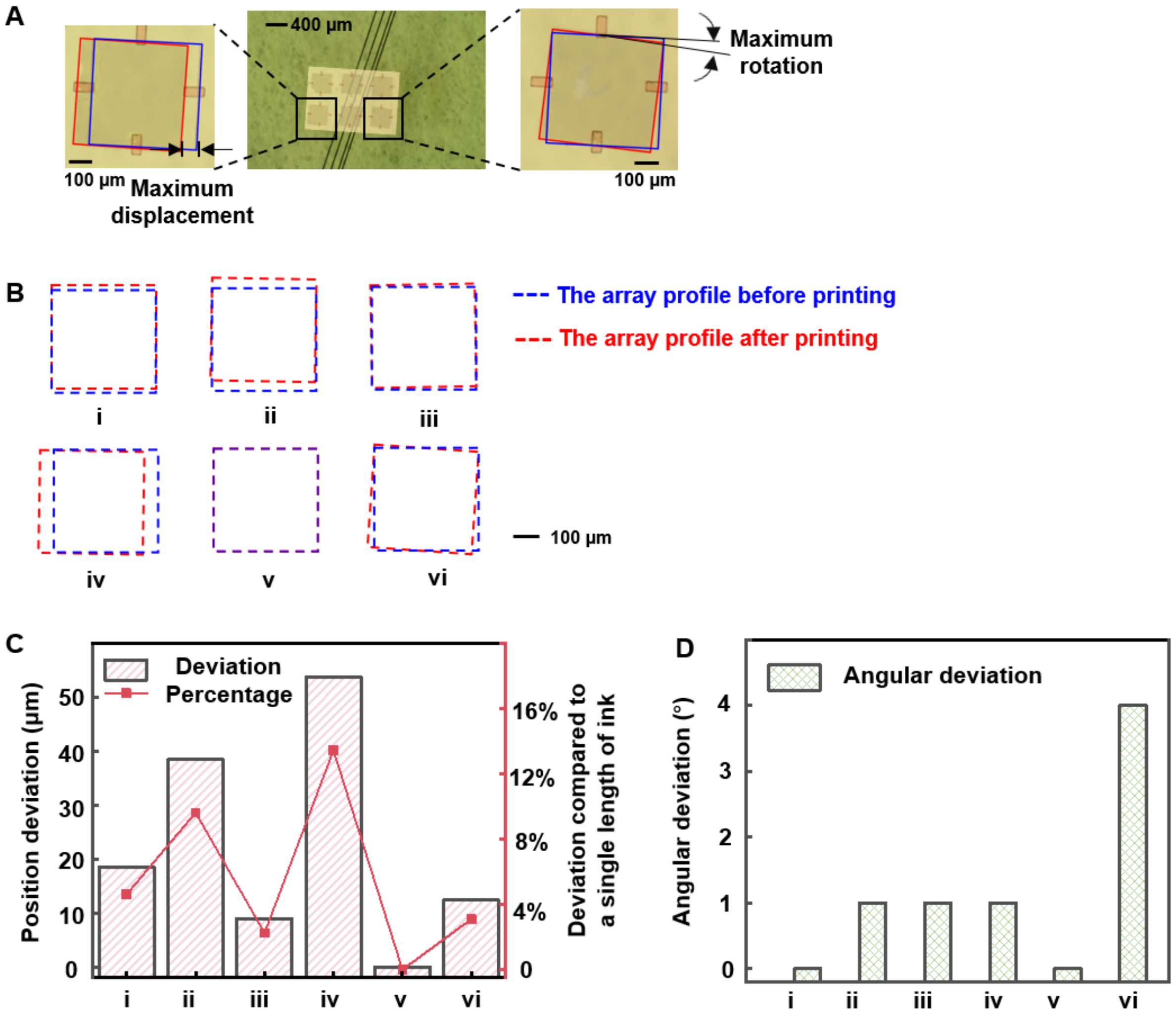

Supplementary Figure 18. (A) Si platelet images overlaid before and after the transfer printing process with the maximum relative displacement and angular rotation labeled. (B) The array profiles of Si platelets before and after the transfer printing process. (C) The center displacement of each platelet and the deviation percentage relative to a single length (400 μm) of ink before and after the transfer printing process. (D) The rotation angle of each platelet before and after the transfer printing process. Source data are provided as a Source Data file.

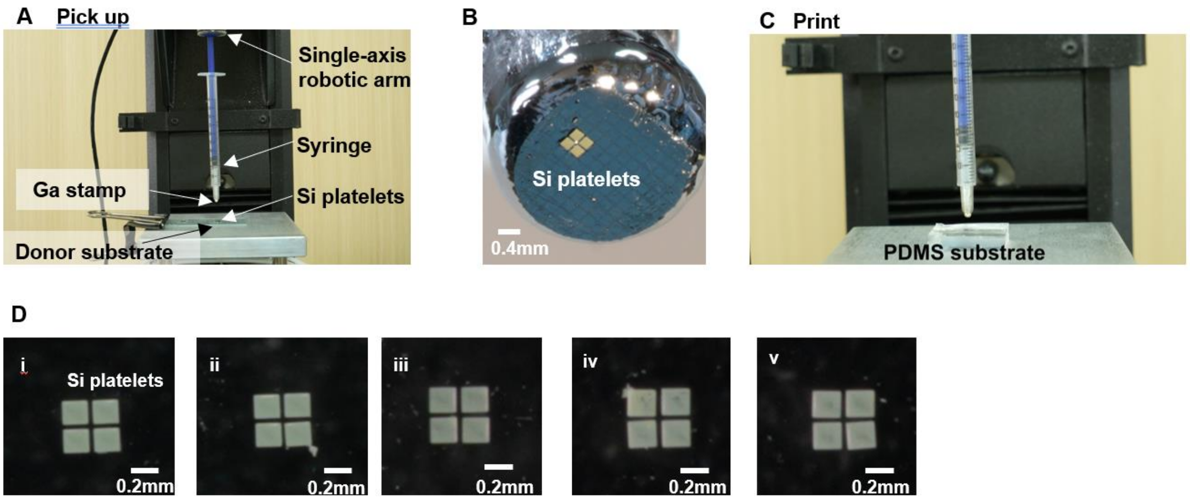

Supplementary Figure 19. (A) The pick-up process of the PLMT method conducted by a single-axis robotic arm. (B) A 2×2 square silicon platelet array (each with dimensions of 200 μm × 200 μm × 2 μm) picked up by a solid Ga stamp. (C) The print process of the PLMT method conducted by a single-axis robotic arm. (D) Five 2×2 square silicon arrays transfer printed by a single-axis robotic arm on a PDMS substrate.

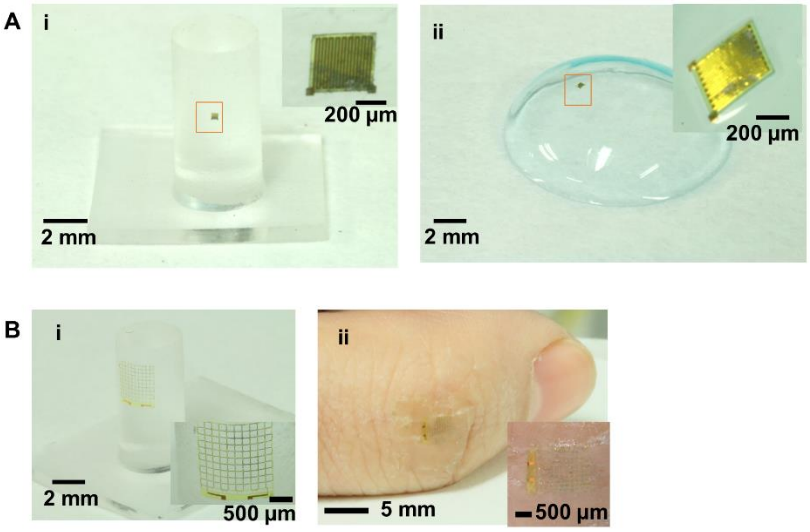

Supplementary Figure 20. (A) Optical images of the flexible resistance sensors prepared on a glass and transfer printed onto (i) a PDMS cylinder and (ii) a contact lens. (B) Optical images of the flexible temperature sensor prepared on a glass and transfer printed onto (i) a PDMS cylinder and (ii) the thumb knuckle in a bent position.

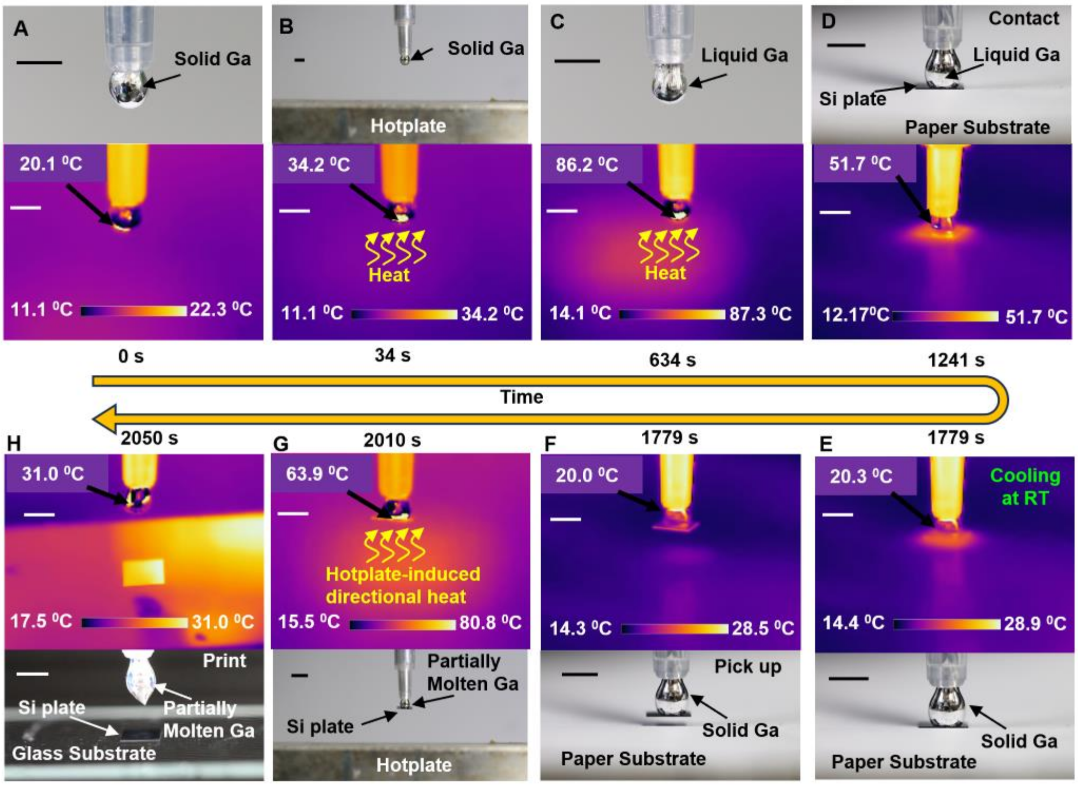

Supplementary Figure 21. Thermograms and optical images of the pickup and printing process through hotplate-induced directional heating to transfer print a macroscale Si plate with 5 mm × 5 mm × 100 µm. Scale bars: 4 mm.

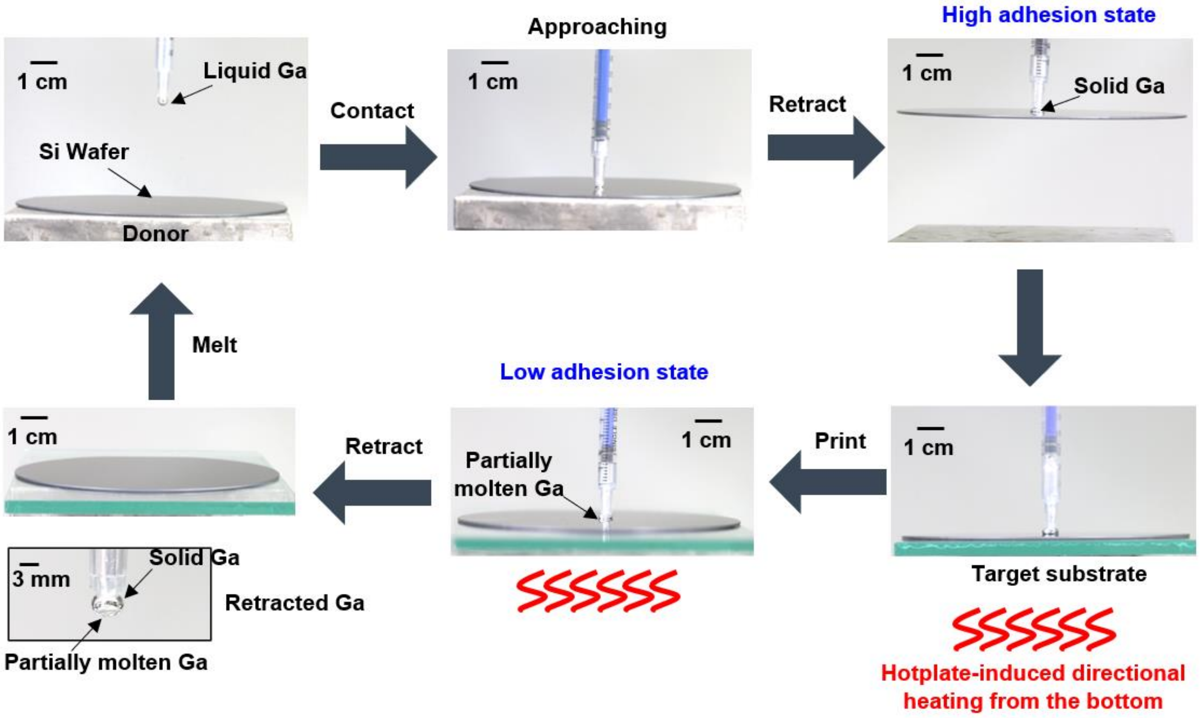

Supplementary Figure 22. Schematic illustration of the pickup and printing process of PLMT to transfer print large Si wafer with a 10 cm diameter.

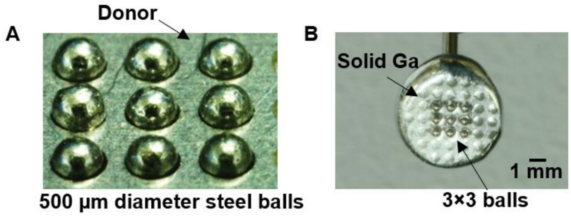

Supplementary Figure 23. A 3 by 3 array of 500 µm diameter steel spheres (A) prepared on a steel donor and (B) picked up and fixed by the solid Ga.

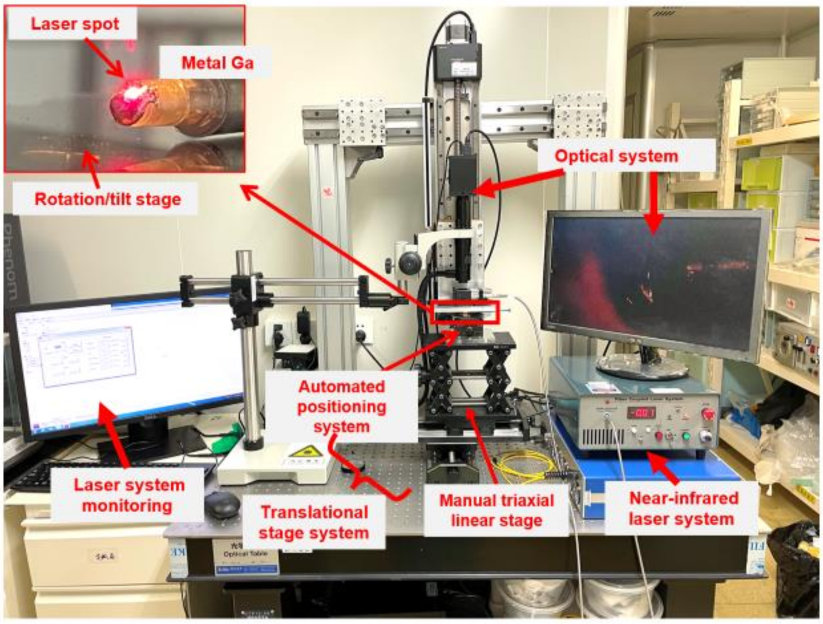

Supplementary Figure 24. Photography of the laser-induced heating system with the translational stages system. The whole system consists of a laser system, an optical system for in-situ monitoring, and the translational stage system for the alignment. The inset illustrates the solid Ga aligned in the system.

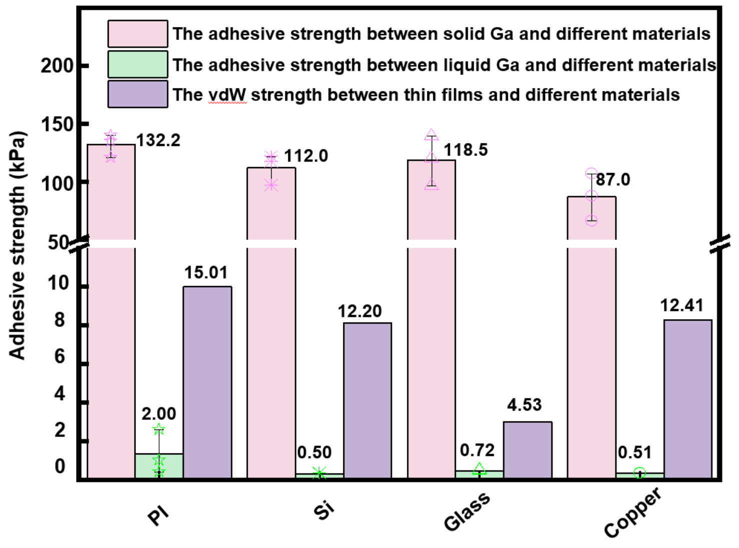

Supplementary Figure 25. The van der Waals (vdW) strength between thin films and different materials compared to the adhesion strength between metal gallium and different materials. The standard deviation is based on 3 repeated experiments. Source data are provided as a Source Data file.
