# Supplementary material for: Precision-induced localized molten liquid metal stamps for damage-free transfer printing of ultrathin membranes and 3D objects
Source: Nat Commun. 2024 Oct 13;15:8839. doi: 10.1038/s41467-024-53184-7 (PMC11471857; doi:10.1038/s41467-024-53184-7)
Supplement: Supplementary file 2 — Description of Additional Supplementary Files [file 41467_2024_53184_MOESM2_ESM.pdf]

**Description of Additional Supplementary Files**

**Supplementary Data 1:** Data file for the initial configuration before the contact process of the Ga-based stamp on the nanoscale silicon wafer.

**Supplementary Data 2:** Data file for the final configuration after the contact process of the Ga-based stamp on the nanoscale silicon wafer.

**Supplementary Data 3:** Data file for the configuration after the crystallization from liquid state to solid state of Ga.

**Supplementary Data 4.** A Matlab code for correlation coefficient analysis.

**Supplementary Movie 1.** The transfer printing of microscale Si platelets (400 μm × 400 μm × 2 μm) using localized molten metal gallium by laser-induced transient heating.

**Supplementary Movie 2.** The extrusion and withdrawal of liquid gallium through the syringe.

**Supplementary Movie 3.** A macroscale Si platelets array transfer printed by localized molten metal gallium through a single-axis robotic arm.

**Supplementary Movie 4.** Macroscale Si plate transfer printed by localized molten metal gallium through hotplate-induced directional heating.
